# Supplementary material for: Novel Exon-Skipping Therapeutic Approach for the DMD Gene Based on Asymptomatic Deletions of Exon 49
Source: Genes (Basel). 2022 Jul 19;13(7):1277. doi: 10.3390/genes13071277 (PMC9323532; doi:10.3390/genes13071277)
Supplement: Supplementary file 1 [file genes-13-01277-s001.zip › genes-1784593-supplementary.pdf]

Supplementary Table S1

Authors	Phenotype	Patients	Genomic Breakpoints(hg19/GRCH37)	Notes
**Banerjee et al.**	DMD	2	Unknown	
**Giliberto et al.**	DMD	1	Unknown	Dystrophin Absent on WB
**Mital et al.**	DMD	3, (DG, KS, RI)	Unknown	we note for KS and RI that southern blots were normal
**Zamani et al.**	DMD	1	Unknown	
**Deepha et al.**	DMD	3 (205,206,210)	Unknown	
	BMD	1 (209)		
**UMD-DMD**	Unknown	3 (ID :17760,17686, 17572)	Unknown	Origins : China
**Brison et al.**	Likely DMD	Family ID: 6	g.(?_31,854,529)_(31,877,889_ ?)	NIPS then verified by CGH array in a male foetus
**Marey et al.**	BMD	1 (Patient #5272)	g.31839857_31892375del	**Only deletion with precise genomic breakpoints**
**Yun et al.**	BMD	2 (ID: 15, 15-1)	Unknown	
**Clinvar (Baylor Genetics)**	BMD	Variant ID : 625643	Unknown	Clinvar (Baylor Genetics)
**Clinvar (invitae)**	DMD	Variant ID : 833063	Unknown	Clinvar (invitae)
**LOVD**	BMD	Individual: 00135563		
	Unknown (Pathogenic?)	Individuals: 00137264,00239877,00297283,00297696,00287744, 00297304, 00297717		

List of reported isolated deletion of exon 49 in literature, in total : 28 isolated deletion of exon 49
